# Whole-Genome Sequences of Five Strains of Kocuria rosea, NCTC2676, NCTC7514, NCTC7512, NCTC7528, and NCTC7511

**DOI:** 10.1128/MRA.00256-19

**Published:** 2019-10-31

**Authors:** Jake D. Turnbull, Julie E. Russell, Mohammed-Abbas Fazal, Nicholas E. Grayson, Ana Deheer-Graham, Karen Oliver, Nancy Holroyd, Julian Parkhill, Sarah Alexander

**Affiliations:** a Culture Collections, Public Health England, London, United Kingdom; b Wellcome Trust Genome Campus, Wellcome Trust Sanger Institute, Hinxton, United Kingdom; University of Maryland School of Medicine

## Abstract

Kocuria rosea is a Gram-positive coccus found in the environment and within normal human skin microbiota, and more recently, it has been potentially implicated as an opportunistic pathogen. Here, we describe the genome sequences of five strains of *K. rosea* (NCTC2676, NCTC7514, NCTC7512, NCTC7528, and NCTC7511).

## ANNOUNCEMENT

Kocuria rosea is a non-spore-forming, aerobic, oxidase-negative, catalase-positive, Gram-positive coccoid bacterium which grows as circular, smooth, pinkish colonies on nutrient agar. This organism is traditionally known as a saprophyte and commonly exists in the natural environment (soil, water, and air) (1). *K. rosea* is also a commensal of human skin and the oropharynx and is very rarely a human pathogen, although due to potential misidentification and micrococci being thought of as contaminants, its role in infection might be underestimated (2). *K. rosea* has been described as an opportunistic pathogen; it has been known to cause medical device-related disease in the immunocompetent and in those with underlying conditions, and it has been implicated in isolated cases of urinary tract infection, bacteremia, endocarditis, and peritonitis in those undergoing peritoneal dialysis (3–6). Phenotypic and biochemical identification techniques may fail to correctly identify *K. rosea*, and genome-based identification methods may be required to build a more complete picture of the disease caused by this organism (2). Currently, there are only three draft genome sequences available for *K. rosea* strains. Here, we report additional genome sequences of five strains deposited in the National Collection of Type Cultures (NCTC). Three of these genomes have been assembled into a single contig.

Due to the historical nature of the strains sequenced in this study, the availability of metadata is limited. NCTC records highlight that all five strains were isolated prior to 1949. Notably, all of them are listed in a study conducted in the NCTC in the late 1940s, the aim of which was to provide a ubiquitously useful system of classification for aerobic catalase-positive Gram-positive cocci by examining 431 strains for morphological characteristics, biochemical characteristics, and sensitivity to two bacteriophages (7). It is also known that NCTC7512, NCTC7514, and NCTC7528 were once a part of the Kral Collection, one of the world’s first microbial culture collections which operated from 1890 until 1911.

The five NCTC strains were recovered from lyophilized ampules, cultured on nutrient agar, and incubated at 37°C for 48 h. Genomic DNA was extracted from the pure cultures using the Qiagen midi kit and a 100/G Genomic-tip (Manchester, UK). Whole-genome sequencing (WGS) was performed using the Pacific Biosciences (PacBio) RS II platform. DNA was sheared to 15 kb, followed by preparation of a 10 kb- to 20-kb library and sequencing utilizing C4/P6 chemistry.

Sequence reads were assembled using HGAP v3 of the SMRT Analysis software v2.3.0 (8). The fold coverage to target when picking the minimum fragment length for assembly was set to 30, and the approximate genome size was set to 3 Mbp. The assembly was circularized using Circlator v1.1.3 (9). Finally, the circularized assembly was polished using the PacBio RS_Resequencing protocol and Quiver v1 of the SMRT Analysis software v2.3.0. Automated annotation was performed using Prokka v1.5 and a genus-specific database from RefSeq (10). The summary statistics of the genomes of the five strains are shown in Table 1.

**TABLE 1:** Summary statistics of the genomes of 5 NCTC strains of Kocuria rosea

NCTC strain no.	BioSample accession no.	Genome size (bp)	G+C content (%)	No. of contigs	Avg read length (bp)	Avg read coverage (×)	No. of sequencing reads	No. of CDS^*a*^	No. of rRNA genes	No. of tRNA genes
NCTC2676	SAMEA37374418	3,261,460	68.8	1	3,733	189	187,257	2,972	6	46
NCTC7514	SAMEA26388418	3,763,037	72.9	1	2,860	133	192,710	3,383	9	48
NCTC7512	SAMEA104200652	3,811,253	72.9	1	3,772	91	115,132	3,389	9	47
NCTC7528	SAMEA26389168	3,936,932	72.6	2	2,927	172	258,620	3,525	9	47
NCTC7511	SAMEA24561418	3,928,081	72.2	4	2,213	56	119,162	3,512	14	47

a CDS, coding sequences.

### Data availability.

The complete genome sequences have been deposited in the National Center for Biotechnology Information under BioProject number PRJEB6403 and BioSample numbers SAMEA37374418, SAMEA26388418, SAMEA104200652, SAMEA26389168, and SAMEA24561418 for strains NCTC2676, NCTC7514, NCTC7512, NCTC7528, and NCTC7511, respectively. The Sequencing Read Archive accession numbers are ERR2125691, ERR2125654, ERR2171932, ERR2125655, and ERR2125642, respectively.
